# Influence of Cooperation Work on Management Continuity of Ambulatory Cardiovascular Care: A Cross-Sectional Exploratory Study in Germany

**DOI:** 10.5334/ijic.7019

**Published:** 2023-12-06

**Authors:** Christine Arnold, Patrick Hennrich, Frank Peters-Klimm, Michel Wensing

**Affiliations:** 1 Department of General Practice and Health Services Research, Heidelberg University Hospital, Germany; 2 Division of Neonatology, Department of Paediatrics, Inselspital, Bern University Hospital, University of Bern, Switzerland

**Keywords:** integrated care, continuity of care, ambulatory cardiovascular care, team climate

## Abstract

**Introduction:**

A wide range of factors influence coordination and continuity of care. The aim of this study was to explore how management continuity of cardiovascular-related ambulatory care is influenced by the following network characteristics: presence of a case coordinator, network reciprocity, network composition and team climate.

**Methods:**

This cross-sectional observational study included three written surveys. The primary outcome management continuity of cardiovascular care was measured with the team/cross-boundary scale in the Nijmegen Continuity Questionnaire. The final analysis comprised a multivariate linear multilevel model with the predictors: presence of a case coordinator, network reciprocity, network composition and team climate.

**Results:**

Eighteen general practices with 83 health workers and 340 patients participated. The linear multilevel regression analysis showed a positive influence of team climate on cross-boundary continuity of care (b-coefficient 0.44, 95% confidence interval 0.09–0.78, p = 0.02). No statistically significant influence was measured for the other predictors.

**Discussion:**

To improve integrated care, therefore, emphasis should also be placed on promoting the team climate within individual practices. Regarding network characteristics, further research is needed, especially in larger practices.

**Conclusion:**

This study showed that team climate had an independent, relevant and statistically significant association with cross-boundary continuity of cardiovascular ambulatory care.

## Introduction

Cardiovascular diseases continue to have a high prevalence and are one of the leading causes of death worldwide. With the increase in multimorbidity, the care of patients with cardiovascular diseases is becoming more complex and requires the integration of various health workers to achieve optimal patient care [1,2]. Optimal patient care requires high continuity of care, which is a central characteristic of effective integrated care models. Integrated care is structured healthcare delivery to a specific population (e.g. coronary heart disease patients), involving various healthcare providers and informed by the best available evidence [3]. Higher continuity of care is associated with lower mortality rates [4,5], lowered hospitalisations [6,7] and increased feelings of security and confidence for the patients [8,9].

Continuity of care has been defined by Haggerty et al. [10] as a three-dimensional concept: informational continuity (information sharing between health workers); relational continuity (trusting relationships with health workers); and management continuity (if multiple health workers are involved in care, their approach is consistent with one another’s and congruent with patient needs) [10,11].

The focus in this study is on management continuity in ambulatory cardiovascular care, which is challenging in healthcare systems that lack clinical guidance that is shared across healthcare providers. Many factors influence provider’s views on clinical management, including vocational training and socialisation, the influence of peers, and other considerations. For example, a previous study with claims data regarding physicians-networks showed that the prescription of a new medication for heart failure is influenced by network structures. When physicians were connected to other physicians through common patients who were already prescribing the new medication, the likelihood of prescribing the new drug increased [12]. However, little is known about the views of healthcare providers are influenced by the teams and networks in which they are embedded. For example, an interview study of hospital admissions and discharges suggests that personal relationships among healthcare providers in different sectors promote alignment of practices [13].

In this study, we focused on the role of ambulatory care providers’ interaction networks. Based on empirical and theoretical work, we developed a conceptual model to explain the impact of professional interaction networks of health workers on care delivery and healthcare outcomes. Health workers conduct various activities, including diagnosis, counselling, treatment and prevention, and these activities must be coordinated. We hypothesized that coordination of care is influenced by the following factors: presence of a case coordinator (typically a generalist physician or nurse [14]), network reciprocity, team climate, and change of network composition. Together these factors influence cooperation work, which covers various processes such as convincement, competition, use of power, and selection of network members.

Over time, network reciprocity within interactions between network members increases mutual trust and reduces risk of defecting behaviours. Research in evolutionary biology has shown that, over time, network reciprocity is crucial for the emergence of altruistic cooperation and that it may even counterbalance short-term individual benefits of non-cooperation behaviours [15]. Under certain conditions, this also applies to anticipated rather than experienced reciprocity.

A moderate change of network composition as opposed to no change makes it possible to select co-operators and unselect non-co-operators, while maintaining the favourable effects of repeated interactions on cooperation [15]. This change is likely to influence the structure of referral and advice networks between primary care and other health professionals. It can also contribute to the homogeneity of network members.

In addition to these structural network factors, a good climate and team culture in teams can enhance management continuity as well as experience of healthcare providers [16]. According to Mundt et al. [17], a shared team vision prevails in small centralised networks, which are associated with higher quality of care and lower costs.

The aim of this study was to explore how management continuity of cardiovascular-related ambulatory care is influenced by the following network characteristics: presence of a case coordinator, network reciprocity, network composition and team climate.

## Methods

The study was conducted in accordance with the Declaration of Helsinki and received approval from the Ethics Committee of the Medical Faculty of Heidelberg (ID: S-726/2018) and from the respective State Medical Chambers. Due to the anonymity of the patient survey, the Ethics Committee of the Medical Faculty of Heidelberg approved a waiver for informed consent. Additionally, participants were informed about this waiver in writing and that returning the questionnaire was sufficient. We registered the study prospectively on 07/11/2019 at the German Clinical Trials Register under the ID: DRKS00019219. The STROBE reporting guideline [18] for observational studies was followed in the reporting of this study.

### Study design and study population

This cross-sectional observational study explored the influence of coordination work on management continuity of cardiovascular care in German primary care and consisted of three written surveys. The three-year (2019–2022) ExKoCare project aimed to recruit a sample of 40 general practices in the German states of Baden-Wuerttemberg (approximately 11 million inhabitants; sampling took place in 10 of the 44 counties), Rhineland-Palatinate (approximately 4 million inhabitants; sampling in 13 of the 36 counties), and Saarland (approximately 1 million inhabitants; sampling in 2 of the 6 counties) [19]. The study included three samples: general practices; their physicians and non-physician health workers; and patients from these general practices with at least three recorded chronic diseases, one of which was coronary heart disease.

### Data collection

#### General practices

We aimed to recruit 40 general practices and anticipated a low participation rate of 5%. The general practices were recruited from a clustered, stratified sample from a total of 25 counties. The counties were chosen with regard to population density to ensure that rural and urban areas were equally represented. We aimed to contact each general practice via fax or email. The owners of each general practice were identified through the publicly available online physician’s databases of the three states. This led to an initial sample of 1,617 practices (Baden-Wuerttemberg, 912 practices; Rhineland-Palatinate, 596 practices; Saarland, 109 practices) [20]. Some basic characteristics of the practices were documented in a practice questionnaire completed by the practice owner. These characteristics included the size of the practice, as measured by the number of cases per quarter, and the participation in case-management or disease-management programmes.

#### Health workers

After the practice owners consented to the ExKoCare study, all health workers over the age of 18 of the general practices were contacted personally in writing and invited (*n* = 208) to participate in the survey. Data were collected using a written pseudonymized questionnaire. All participants gave written informed consent for the study.

#### Patients

After the health worker survey was completed, all participating practices were contacted and invited to support patient recruitment. Initially, the aim was to recruit 15 patients from each general practice (*n* = 600). Based on previous research, we expected a response rate of 30%, and so we intended to invite 50 patients per practice. The research team assisted the practices via phone in identifying potential study participants to ensure that the inclusion criteria (adult patients with three or more chronic conditions, including at least one atherosclerosis-related cardiovascular condition, only adult patients over the age of 18 are eligible to participate in this study, ability to consent to participate) were met. A list of potential participants was compiled from the physicians’ billing systems. This system lists all patients who have been billed for services, regardless of whether they regularly visit this doctor. Then, up to 50 patients were selected by selecting every 3rd patient from a starting point specified by the researcher. If there were fewer than 50 patients on the list, all were selected. Before this selection, the physician was asked to check for the cognitive ability to complete a questionnaire and potential contra-indications. Patients were sent the questionnaire by post, and an anonymous return envelope addressed to the research department was included, so that the practices did not know which patients participated.

### Measures

For each stage of the study, we used a newly developed questionnaire that included both validated and newly created parts derived from previous studies and guidelines. The three questionnaires used a pseudonym for each practice, so that the results could be assigned accordingly.

#### Outcome

The outcome of the study was management continuity of cardiovascular care, which was measured from the patient’s perspective with the team/cross-boundary scale in the Nijmegen Continuity Questionnaire (NCQ) [21,22]. The NCQ was developed in the Netherlands and has been validated in various countries and applied in different settings (e.g., in Norway in the field of rehabilitation) [23]. The 12-item NCQ includes the following subscales: personal continuity-1 (‘care provider knows me’, items 1–5), personal continuity-2 (‘care provider shows commitment’, items 6–8), and team/cross-boundary continuity (items 9–12). The items 9–12 include the following statements: “These care providers transfer information very well to each other.”, “These care providers work together very well.”, “The care of these care providers is very well connected.” and “These care providers always know very well from each other what they do.” In the ExKoCare project, the two subscales for personal continuity were used to assess the continuity regarding the family doctor and cardiologist, the team part of the team/cross-boundary subscale was used to assess the continuity in the general practice (continuity between physicians and practice assistants) and the cross-boundary part was used to assess the continuity between family doctor and cardiologist. Thus, the questionnaire contained six continuity of care scores and a total of 24 items. Each question could be answered on a 5-point Likert-scale (1 = *strongly disagree*, 2 = *disagree*, 3 = *neutral*, 4 = *agree* to 5 = *strongly agree*) or with *I do not know*.

The English version of all 12 items in the NCQ was translated carefully and independently by CA and PH using a forward translation into German and a backwards translation into English. After the independent translations, consensus discussions were held and a common German version was produced. A test involving interviews with six patients did not identify any significant lack of clarity [24]. For this part of the project, we used only the scales for team and cross-boundary continuity, which served as the outcomes of the study. For the calculation of the two outcomes, one missing value was allowed in each case.

#### Predictors

*Presence of care coordinator* was measured in the practice questionnaire using a dichotomous item (yes/no) that indicated the presence of a case manager for cardiovascular care.

*Reciprocity* indicates the percentage of returned relationships in a directed network. The value range is between 0 and 1. The network was constructed based on the questionnaire in which the health workers were asked about the weekly exchange of information within their general practice. They were asked to mark the persons with whom they exchange information weekly [25].

*Change of network composition* was measured on a 5-point Likert scale, with 1 indicating little change in the last two years regarding the collaboration with cardiologists outside the health worker’s own general practice and 5 indicating a high level of change. This predictor was also used to measure network changes within the general practice and among health workers outside the general practice.

*Team climate* within the general practice was measured with the short version of the Team Climate Inventory scale [26]. This questionnaire contains 14 items and four subscales: 1) clear and realistic objectives to which the team members are committed (called ‘shared vision’, 4 items); 2) interaction between team members (called ‘psychological safety’, 4 items); 3) commitment to high standards of performance and appraisal of weakness (called ‘task orientation’, 3 items); and 4) support for innovation (called ‘innovation support’, 3 items). The responses were given on a 5-point Likert scale from 1 = *disagree* to 5 = *agree*. We translated the questionnaire into German using the forward-backward method, which was completed in each part by two individuals. For each participant, the mean of the 14 items was calculated. One missing value was permitted. The mean value for each practice was then determined. Higher scores indicated a better team climate. Thus, a mean with a standard deviation for each practice was available with a possible range of 1 to 5.

#### Potential confounders

We included *network size* that indicates the number of health workers in each general practice. In addition, the number of physicians and non-physician professionals was measured. From the patient questionnaire, we included the number of chronic diseases (from a list of ten).

### Statistical analyses

First, we conducted a descriptive analysis of the study population. According to the measurement level, relative and absolute frequencies and mean values with *SD*s were calculated. Next, descriptive statistics were calculated for the predictors, and then the patient-reported outcome and team and cross-boundary continuity of care with means and *SD*s were calculated. The final analysis comprised univariate and multivariate linear multilevel regression analyses. Before this, the independent variables were tested for multicollinearity, which was assumed when the correlation coefficient was larger than 0.6. The two final models included the following predictors: presence of care coordinator (*yes* or *no*), reciprocity (range 0–1), change of network composition (range 1–5) and team climate (range 1–5), which should express the cooperation work. Additionally, the models were adjusted for network size and number of chronic diseases. In the first model, the dependent variable was the cross-boundary continuity of care between general practice and cardiologist practice; in the second model, it was team continuity of care within the general practice. Based on the nested data in GP practices, the interclass correlation coefficient was calculated to compute the proportion of variance explained by the GP practice in the total variance. All analyses were performed with the statistics software R (version 4.0.2) using RStudio (version 1.2.5033). The significance level was set at an alpha of 0.05.

## Results

Data were collected from November 2019 to December 2021 (health workers survey from November 2019 and January 2021 and patients from November 2020 to December 2021), that was predominantly during the COVID-19 pandemic. After exclusion due to unavailable fax numbers or incorrect deliveries, we contacted 1,511 family practices, from which 42 took part in the ExKoCare project (response rate 2.8%). Eighteen general practices had collected all data for this study and were included in the present analysis. Due to the increased workload during the COVID-19 pandemic, the data from the other practices (*n* = 24) were not complete, which led to their exclusion from this analysis. From the 18 practices, a total of 93 health workers and 596 patients were invited to participate in the study. With response rates of 89.3% and 57.0%, respectively, 83 health workers and 340 patients were included in the study. Table 1 provides an overview of the characteristics of the participating practices and patients. Over 75% of the practices were single-handed practices with an average of 1.4 physicians and 3.8 non-physician health workers. The patients were on average 74.5 years old (range 45–94), and 247 (72.9%) participants were male.

**Table 1:** Practice and patient characteristics.


*PRACTICE CHARACTERISTICS*	N = 18

**Type of practice**, n (%)	

Not single-handed practice	4 (22.2)

Single-handed practice	14 (77.8)

**Total number of health workers**, mean (SD)	5.2 (1.2)

Physicians, mean (SD)	1.4 (0.5)

Non-physicians, mean (SD)	3.8 (1.0)

**Cases per quarter**, n (%)	

<500 cases	0 (0.0)

500–1,000 cases	7 (38.9)

1,001–1,500 cases	7 (38.9)

>1,500 cases	4 (22.2)

***PATIENT CHARACTERISTICS***	**N = 340**

**Continuity of care**	

Team, mean (SD)	4.0 (0.7)

Cross-boundary, mean (SD)	3.8 (0.8)

**Age**, mean (SD), *n = 316*	74.5 (9.1)

**Sex**, n (%), *n = 339*	

Female	92 (27.1)

Male	247 (72.9)

**Number of chronic diseases**, mean (SD),	1.6 (1.5)

**Participation in DMP**, n (%)	76 (24.8)


SD = Standard deviation. DMP = Disease management programme.

The practices examined had an average network size of 5.2 (*SD* 1.2). In addition to the measured network characteristics (Table 2), the team climate score across all practices was on average 4.2 (*SD* 0.4). With a maximum value of 1, the average network reciprocity was 0.6 (*SD* 0.3). Patients (*n* = 231) reported cross-boundary continuity of care between family doctor and cardiologist with a mean of 3.8 (*SD* 0.8) and team continuity of care within the general practice (*n* = 270) with a mean of 4.0 (*SD* 0.7).

**Table 2:** Descriptive data of network characteristics.


*NETWORK CHARACTERISTICS*	MEAN (SD)

	N = 18

**Presence of case coordinator**, *n (%)*	*n = 17*

No	12 (70.6)

Yes	5 (29.4)

**Network reciprocity**	0.6 (0.3)

**Change of network composition**	

Changes outside the general practice	1.7 (0.7)

Changes inside the general practice	2.2 (0.9)

Changes cardiology practice	1.6 (0.6)


In the univariate regression analyses, only team climate and the number of chronic conditions showed a statistically significant impact. Therefore, we excluded the confounders age and sex from the final model. The linear multilevel regression analysis showed a positive influence of team climate on cross-boundary continuity of care (*b*-coefficient 0.44, 95% CI 0.09; 0.78, *p* = 0.02). No statistically significant influence was measured for the other predictors (see Table 3). The interclass correlation coefficient for clustering within practices was 0.02. In addition, the predictors in the model with the outcome team continuity of care within general practice were not statistically significant.

**Table 3:** Influence of cooperation work on cross-boundary continuity of cardiovascular care.


	*B*-COEFFICIENT	*SE*	95% CI	*P*-VALUE

Presence of care coordinator (ref. no)	0.08	0.11	–0.16; 0.32	0.50

Reciprocity	–0.21	0.20	–0.65; 0.22	0.31

Change of network composition	–0.05	0.08	–0.23; 0.13	0.56

Team climate	0.44	0.16	0.09; 0.78	0.02^*^

Intercept	2.07	0.73	0.63; 3.51	<0.01


*n* = 231 patients and 17 general practices. Model adjusted for network size and number of chronic diseases. * Statistically significant at α = 0.05. ref. = reference group, CI = confidence interval, SE = standard error.

## Discussion

This exploratory study found a positive influence of the team climate within general practices on patient-reported cross-boundary continuity of cardiovascular ambulatory care. However, no effect of the hypothesized network mechanisms was found. In addition, team climate did not have a statistically significant impact on team continuity of care within the general practice. It may also be noted that German primary care practices are often small, so a practice typically has one team.

Previous studies have shown that team climate positively influences interprofessional collaboration, which is important for managing continuity of care [27]. While within the practice it may be possible to compensate for a lower team climate, it seems to be even more important that there is a good team climate within the practice in order to achieve cross-boundary continuity of care, which may explain the different results in the two measured team and cross-boundary continuity of care scores.

Outside of the practice, various arrangements must be made in which tasks may not be precisely assigned to practice members, making coordination more difficult, possibly leading to fewer agreements if the team climate is poorer and, ultimately, reducing the continuity of care.

Promoting the team climate in individual practices could therefore also contribute to improving integrated care. However, we are not aware of any studies that have examined the relationship between team climate and continuity of care in ambulatory practices.

The network mechanisms, which influence cooperation work, studied provided a first indication of influence on cross-boundary continuity of care, but the influence was not statistically significant.

### Limitations

The study has limitations in terms of unknown measurement validity of several predictors and generalisability due to sampling procedures, so the descriptive figures should be carefully interpreted despite the use of a validated outcome measure. As the data collection took place during the corona pandemic, this could be of influence on the nature of the collected data and the low response rate. It follows that the findings should be interpreted as preliminary.

A weakness of the study is the relatively high non-response in the team/cross-boundary continuity of care items. For example, of the participating patients, only 231 (68%) completed the questions about cross-boundary continuity of cardiovascular care. A further issue is that only few confounders could be included in the analysis due to the sample size. It is therefore important to conduct further research with larger samples to demonstrate effects of network mechanisms on coordination work.

The findings suggest, that especially in smaller single-handed practices the network structures play a subordinate role compared to the team functioning, since all interact frequently and there is little explicit coordination. It is possible that we would have been more likely to find a statistically significant effect of network mechanisms in a sample with larger practices.

A specific issue is that single-handed practices constituted almost 80% of the sample of practices. Although Germany has a high proportion (60%) of individual practices [28], group practices are increasing due to rising costs for individual practices, and so increasing numbers of practice rooms and staff are shared. The number of practice members is expected to increase, and the structures of the practices will become more important.

Another limitation is that the team climate questionnaire was not explicitly validated for the setting due to low response rate.

## Conclusion

This exploratory study showed that team climate had an independent, relevant and statistically significant association with cross-boundary continuity of cardiovascular ambulatory care. The hypotheses regarding network structures were not confirmed, but it can be supposed that the structures and division of tasks in practices are becoming increasingly important due to the expansion resulting from mergers of single-handed practices.
